# Case Report: Effective IL-4/IL-13 axis suppression and resolution of severe atopic dermatitis by dupilumab monotherapy in a patient with IPEX syndrome

**DOI:** 10.3389/fimmu.2026.1923415

**Published:** 2026-08-17

**Authors:** Mohammed Alshanti, Matthew S. MacDougall, Merve Nida Gokbak, Akshaya Ramachandran, Amna Aref Alzaabi, Jessie L. Alexander, Rosa Bacchetta, Hiba Mohammed Shendi

**Affiliations:** 1 Division of Allergy and Immunology, Department of Pediatrics, Sheikh Khalifa Medical City, Abu Dhabi, United Arab Emirates; 2 Division of Allergy, Immunology, Rheumatology, Stanford University, Stanford, CA, United States; 3 Division of Stem Cell Transplantation and Regenerative Medicine, Department of Pediatrics, Stanford University, Stanford, CA, United States; 4 Division of Dermatology, Department of Medicine, Sheikh Khalifa Medical City, Abu Dhabi, United Arab Emirates

**Keywords:** dupilumab, FOXP3, inborn error of immunity, IPEX, type 2 immunity

## Abstract

**Introduction:**

The treatment of immune dysregulation, polyendocrinopathy, enteropathy, X-linked (IPEX) syndrome has largely focused on immunosuppression with little impact on the atopic manifestations of the disease. Dupilumab therapy has been used successfully in primary atopic diseases and immune regulatory disorders to control type 2 inflammation. Indeed, it has been reported in two prior IPEX cases, but in combination with immunosuppressive agents. Here, we are the first to report the clinical and immunologic impact of dupilumab monotherapy in IPEX.

**Case:**

A 4-year-old boy with a confirmed hemizygous Forkhead box protein 3 (FOXP3) missense variant (c.1150G>A; p.Ala384Thr) presented with severe, treatment-refractory atopic dermatitis beginning in the newborn period, failure to thrive, lymphadenopathy, food and environmental allergies, asthma, recurrent febrile neutropenia, and type 1 diabetes mellitus, with a peak IgE of 74,625 IU/mL. Following initiation of dupilumab monotherapy at age 2, the patient had a marked improvement in atopic dermatitis by Eczema Area and Severity Index (EASI) score, a rapid decline in serum IgE, improved growth, reduced respiratory hospitalizations, and a profoundly improved quality of life.

**Results:**

Immunologically, dupilumab effectively suppressed IgE and plasma interleukin-4 (IL-4) and IL-13 to healthy control levels. In addition, C-C motif chemokine ligand 27 (CCL27), important for cutaneous T-cell trafficking, was detected at healthy control levels. Upstream alarmins, eosinophil mediators, T helper 1 (Th1) cytokines, and Treg-specific demethylated region (TSDR) demethylation remained elevated—indicating ongoing systemic immune dysregulation not fully addressed by dupilumab alone.

**Conclusion:**

This case demonstrates that dupilumab monotherapy can effectively suppress the IL-4/IL-13 axis and substantially improve atopic manifestations in IPEX, potentially by restoring skin-specific immune tolerance through rebalancing cutaneous T-cell trafficking. These findings support dupilumab as a targeted adjunctive therapy for the atopic features of IPEX and provide a rationale for the systematic study of dupilumab alone or in combination with other immunosuppression regimens.

## Introduction

Immune dysregulation, polyendocrinopathy, enteropathy, X-linked (IPEX) syndrome is an inborn error of immunity defined by hemizygous mutations in Forkhead box protein 3 (FOXP3) leading to defective regulatory T cell (Treg) function (1). As FOXP3 is also transiently induced in activated effector T cells (Teff) to fine-tune immune responses, its disruption in IPEX impacts both Treg stability and Teff cell regulation, collectively driving the profound immune dysregulation characteristic of the disease. The clinical features are heterogeneous, with multiple autoimmune manifestations including enteropathy and type 1 diabetes mellitus (T1DM). Severe atopic dermatitis is a frequent manifestation associated with type 2 immune response, elevated IgE, and eosinophils (1).

The therapeutic choices for IPEX include pharmacologic immunosuppression (IS) and hematopoietic stem cell transplantation (HSCT) (2). Additionally, a gene therapy strategy has recently been developed and is under evaluation in a Phase 1 clinical trial (NCT05241444). Immune modulation is best achieved with direct mTOR inhibition with sirolimus, which selectively suppresses Teff cells while partially restoring Treg function (2). Broader IS is also utilized, including corticosteroids, calcineurin inhibitors, Janus kinase inhibitors, CTLA4-Ig, mycophenolate, methotrexate, and azathioprine. Often, multiple IS medications are used in combination to optimize efficacy, but at the cost of increased adverse effects. Overall, pharmacological IS can temper disease progression, but it does not fully control ongoing immune dysregulation with heightened type 2 immune responses (3). Notably, increased production of interleukin-13 (IL-13) has been observed in patients’ plasma and in FOXP3 knockout Treg cells (4).

Dupilumab is a biologic that modulates type 2 immunity by blocking the binding of IL-4/IL-13 to the IL-4 receptor alpha (IL-4Rα), leading to marked improvement in atopic diseases. Previously, two patients with IPEX syndrome have been treated with dupilumab in combination with other IS regimens. The first patient with severe enteropathy resembling eosinophilic gastrointestinal disease and atopic dermatitis rapidly improved with dupilumab, steroids, and 6-mercaptopurine (5). Similarly, a case of IPEX with persistent atopic dermatitis improved on dupilumab in combination with sirolimus and prednisone (6). Herein, we present an opportunity to examine the effect of dupilumab monotherapy in a patient with IPEX presented early with severe atopic dermatitis.

## Case description and patient perspective

**Figure 1:**
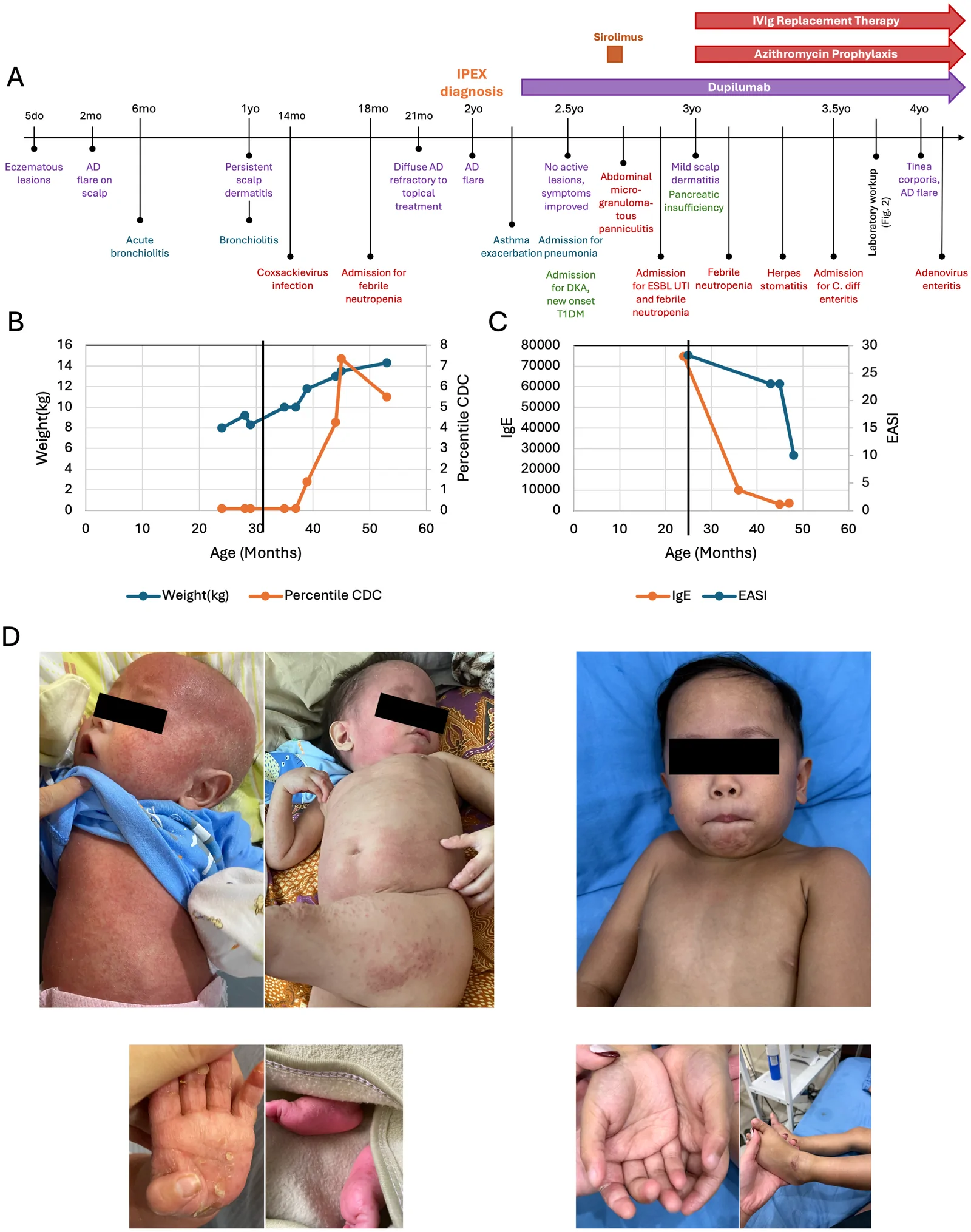
Clinical trajectory of the patient. **(A)** Timeline of atopic (purple), respiratory (blue), infectious (red), and miscellaneous (green) manifestations with initiation of dupilumab, sirolimus, and intravenous immunoglobulin (IVIg) replacement therapy. **(B)** Improved growth trajectory, **(C)** reduction in serum IgE levels and Eczema Area and Severity Index (EASI), and **(D)** marked improvement in cutaneous disease (left, before; right, after) following dupilumab treatment. AD, atopic dermatitis; DKA, diabetic ketoacidosis; ESBL UTI, extended-spectrum beta-lactamase urinary tract infection; *C. diff*, *Clostridioides difficile*; CDC, Centers for Disease Control and Prevention.

A 4-year-old boy, born at 37 weeks to healthy, non-consanguineous parents, diagnosed with IPEX syndrome at the age of 2 presented with multi-system immune dysregulation with severe atopic dermatitis, failure to thrive, lymphadenopathy, food and environmental allergies, asthma, recurrent febrile neutropenia, and onset of T1DM at age 2 (**Figure 1A**). He suffered from frequent viral infections of skin and respiratory and gastrointestinal systems. Baseline immunologic evaluation demonstrated normal serum IgA, IgM, and IgG levels, with lymphocyte subsets within reference ranges. Severe atopic dermatitis began in the newborn period with a peak IgE of 74,625 IU/mL accompanied by asthma and food allergy. His dermatitis quickly progressed to diffuse involvement, remaining refractory to bleach baths, topical corticosteroids, topical tacrolimus, and antifungals. He was diagnosed with IPEX syndrome at age 2, confirmed by a hemizygous, pathogenic FOXP3 missense variant, c.1150G>A (p.Ala384Thr). A second variant in CASP10 (c.1045del; p.Asp349ThrfsTerB) has been associated with autoimmune lymphoproliferative syndrome (ALPS), but our patient had no clinical features of ALPS, and CASP10 was recently shown to be dispensable for FAS-mediated apoptosis (7).

After starting dupilumab 200 mg every 4 weeks at age 2 and ~8 kg, his growth trajectory improved (**Figure 1B**), his skin disease markedly improved as demonstrated by Eczema Area and Severity Index (EASI) score improvement (**Figure 1C**) and IgE rapidly decreased (**Figure 1D**). Since starting dupilumab, he has had minimal atopic dermatitis flares managed with topical corticosteroid. Notably, his dupilumab dosing has not been escalated to 300 mg because he does not meet weight-based criteria. His respiratory course included infant bronchiolitis requiring intubation and persistent asthma on controller therapy with an overall decrease in respiratory admissions after initiation of dupilumab from three to zero. Briefly, prior to dupilumab, the three admissions required bronchodilators with one requiring intubation and mechanical ventilation. Following 5 months of dupilumab, he had one respiratory admission for infection without asthma-related symptoms, suggesting improved control. From the patient and family perspective, treatment with dupilumab profoundly improved quality of life and sleep after initiation. They specifically reported that his difficult-to-manage atopic dermatitis had dramatic and rapid improvement in terms of itch and discomfort. They also reported less asthma-related symptoms and less severity of exacerbations with infection. The patient remained adherent to dupilumab and tolerated it well, with no adverse events reported based on caregiver report, treatment records, and serial clinical assessments. Notably, he was briefly on sirolimus for approximately 2 weeks, and it was discontinued because of abdominal lumps. Biopsy showed a panniculitis-like pattern with non-necrotizing microgranulomas and microabscesses, and the lesions resolved after clindamycin treatment. Given that he was not on any other immunosuppression regimen and only on prophylaxis with immunoglobulin replacement and azithromycin, we consider this an effective result of dupilumab monotherapy. Therefore, dupilumab alone can improve the atopic manifestations of IPEX.

## Diagnostic assessment and discussion

**Figure 2:**
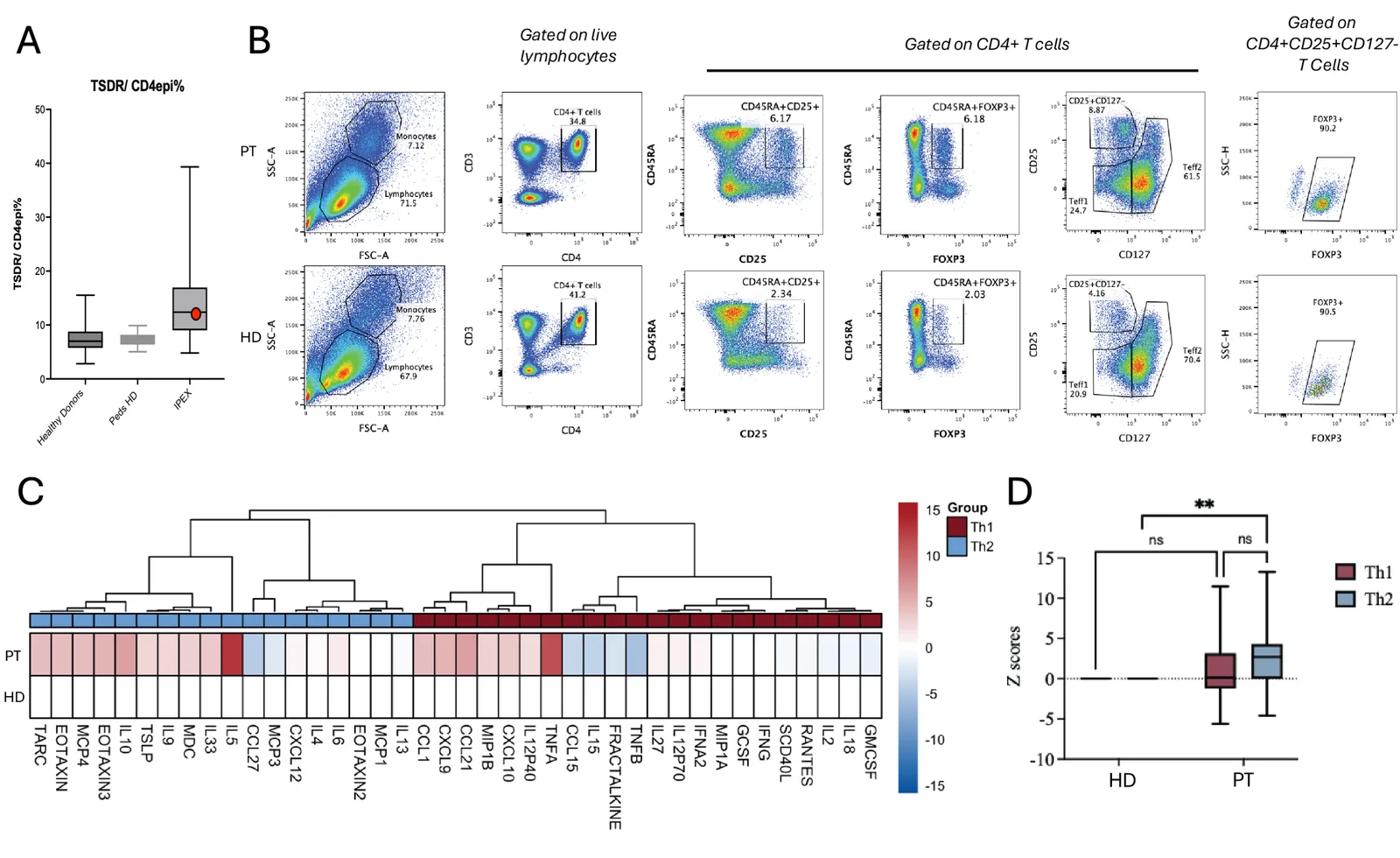
Immunologic and cytokine evaluation of a patient with IPEX at age 3 years 10 months. **(A)** Increased TSDR% of CD4 in our patient (red dot) relative to healthy donors (*n* = 53), pediatric healthy donors (Peds HD) (*n* = 12), and patients with IPEX (*n* = 49). **(B)** Immunophenotyping demonstrates normal frequency of CD4^+^CD25^high^CD127^low^ Tregs with preserved FOXP3 expression. **(C)** Plasma analysis by Luminex shows normal interleukin-4 (IL-4) and IL-13, but elevated eotaxin, eotaxin-3, Thymic stromal lymphopoietin (TSLP), IL-5, tumor necrosis factor alpha (TNFα), and C-X-C motif chemokine ligand 9 (CXCL9), with reduced C-C motif chemokine ligand 27 (CCL27). **(D)** (**: p value= 0.087; ns: not significant). HD, healthy donors; PT, patient.

To objectively assess disease immune status, we evaluated the frequency of Treg-specific demethylated region (TSDR) demethylated cells within CD4-positive cells, the Treg immune phenotype, and plasma cytokines. While on dupilumab monotherapy, our patient had increased frequency of TSDR demethylated cells, which has been demonstrated to be a hallmark of active IPEX disease (**Figure 2A**), despite normal CD4^+^CD25^high^CD127^low^ Treg percentage with preserved FOXP3 expression normally distributed among naïve and memory CD4^+^ T-cell compartments (**Figure 2B**).

Several inflammatory cytokines were elevated in our patient with IPEX unlike healthy pediatric patients including macrophage-derived chemokine (MDC), thymus and activation-regulated chemokine (TARC), C-C motif chemokine ligand 15 (CCL15), IL-13, C-X-C motif chemokine ligand 9 (CXCL9), and CCL1. Patients with IPEX on IS often remain biochemically inflamed with elevated chemokines and cytokines associated with macrophage activation and T helper 2 (Th2) polarization (3). As discussed previously, dupilumab therapy effectively decreased serum IgE, suggesting robust suppression of IL-4/IL-13 signaling. Indeed, IL-4/IL-13 levels were in healthy control range without impact on upstream alarmins (thymic stromal lymphopoietin and IL-33) or eosinophil mobilization and recruitment, reflected by elevated IL-5 and eotaxins (**Figure 2C**). Th2 immunity remained elevated above healthy donors (**Figure 2D**), consistent with the effect of dupilumab in other atopic patients with eosinophilia (8). Importantly, C-C motif chemokine ligand 27 (CCL27), known to be important for T-cell skin homing, was similar to healthy control levels while on therapy (9). Previously, dupilumab has been reported to lead to the expansion of cutaneous Tregs (10). These findings suggest that dupilumab may support restoration of skin-specific tolerance in IPEX by rebalancing cutaneous T-cell trafficking, while Treg trafficking cytokines MDC and TARC remain elevated. Outside of Th2 immunity, dupilumab did not impact the known elevation of Th1 cytokines in IPEX including tumor necrosis factor alpha (TNFα) and CXCL9, though overall Th1 immunity was not significantly increased compared to healthy donors (**Figures 2C, D**).

In conclusion, this patient had improved atopic manifestations while on dupilumab; however, the multi-organ pathology of IPEX makes it challenging to determine whether dupilumab is disease-modifying beyond the atopic manifestations. Our case offered an opportunity to evaluate the effect of dupilumab monotherapy in the context of IPEX immune dysregulation and demonstrated effective suppression of the IL-4/IL-13 axis of type 2 immunity, presenting the possibility of improved immune modulation and disease control. This work is limited in that it is a single case with a single measure at a stable post-treatment time point, limiting conclusions about changes over time. That said, taken together with two prior reports of dupilumab used in combination with other IS regimens (5, 6), dupilumab emerges as an appealing adjunctive and targeted therapy in IPEX, demonstrating benefit for dominant atopic manifestations. These preliminary observations provide a rationale for further systematic studies on dupilumab alone or in combination with other IS in the management of IPEX syndrome.

## Data Availability

The datasets presented in this article are not readily available because the patient’s biologic data was analyzed as part of an ongoing clinical trial. Requests to access the datasets should be directed to rosab@stanford.edu.
